# First description of a lesion in the upper digestive mucosa associated with a novel gammaherpesvirus in a striped dolphin (*Stenella coeruleoalba*) stranded in the Western Mediterranean Sea

**DOI:** 10.1186/s12917-023-03677-2

**Published:** 2023-08-10

**Authors:** Ignacio Vargas-Castro, José Luis Crespo-Picazo, Mª Ángeles Jiménez Martínez, Vicente Marco-Cabedo, Marta Muñoz-Baquero, Daniel García-Párraga, José Manuel Sánchez-Vizcaíno

**Affiliations:** 1 VISAVET Center and Animal Health Department, Veterinary School, Complutense University of Madrid, Madrid, 28040 Spain; 2 Research Department, Fundación Oceanogràfic de la Comunidad Valenciana, 46013 Valencia, Spain; 3 Department of Animal Medicine and Surgery, Veterinary Faculty, Complutense University of Madrid, Madrid, 28040 Spain; 4 Biology Department, Oceanogràfic, Ciudad de las Artes y las Ciencias, 46013 Valencia, Spain

**Keywords:** Herpesvirus, Proliferative lesion, Upper digestive tract, Oropharynx, Laryngopharix, *Brucella*, Transmission, Immunosuppression

## Abstract

**Background:**

A wide variety of lesions have been associated with herpesvirus in cetaceans. However, descriptions of herpesvirus infections in the digestive system of cetaceans are scarce.

**Case report:**

A young female striped dolphin stranded in the Valencian Community (Spain) on the 6th August 2021. The animal showed external macroscopic lesions suggestive of an aggressive interaction with bottlenose dolphins (rake marks in the epidermis). Internally, the main findings included congestion of the central nervous system and multiple, well-defined, whitish, irregularly shaped, proliferative lesions on the oropharyngeal and laryngopharyngeal mucosa. Histopathology revealed lymphoplasmacytic and histiocytic meningoencephalitis, consistent with neuro brucellosis. The oropharyngeal and laryngopharyngeal plaques were comprised histologically of focally extensive epithelial hyperplasia. As part of the health surveillance program tissue samples were tested for cetacean morbillivirus using a real-time reverse transcription-PCR, for *Brucella* spp. using a real-time PCR, and for herpesvirus using a conventional nested PCR. All samples were negative for cetacean morbillivirus; molecular positivity for *Brucella* spp. was obtained in pharyngeal tonsils and cerebrospinal fluid; herpesvirus was detected in a proliferative lesion in the upper digestive mucosa. Phylogenetic analysis showed that the herpesvirus sequence was included in the *Gammaherpesvirinae* subfamily. This novel sequence showed the greatest identity with other Herpesvirus sequences detected in skin, pharyngeal and genital lesions in five different species.

**Conclusions:**

To the best of the authors’ knowledge, this is the first report of a proliferative lesion in the upper digestive mucosa associated with gammaherpesvirus posititvity in a striped dolphin (*Stenella coeruleoalba*).

## Background

Herpesvirus (HV) infections have been observed in mammals, birds, reptiles, fish, amphibians, and bivalves [1]. *Herpesviridae* family consists of three subfamilies: *Alpha*-, *Beta*-, and *Gammaherpesvirinae* [2]. Cetacean herpesvirus strains are usually classified according to the partial nucleotide se quence of a locus of their DNA polymerase (DNApol) gene [3]. So far, only *Alpha*- and/or *Gammaherpesvirinae* have been identified in eight cetacean families: Delphinidae, Kogiidae, Ziphiidae, Physeteridae, Monodontidae, Phocoenidae, Iniidae (odontocetes), and Balaenopteridae (mysticetes) [4–10].

HV can establish latent infection, during which no viral particles are produced [11, 12], and revert to an active replication under stress or immunosuppression [13]. Interestingly, HV also can cause immunosuppression in humans [14, 15], and in other animal species [16–18], including cetaceans [5]. For this reason, it is common to find coinfections with other pathogens, such as Cetacean Morbillivirus (CeMV) [19–22] and *Brucella* spp [20, 22].

In cetaceans, HV molecular positivity has been associated with or without pathological findings [19, 23, 24]. Specifically, Gammaherpesvirus (GHV) positivity have been associated with cutaneous and mucosal lesions, mainly from genital mucosa [4, 6, 19, 24–32]. Recently, GHV infections have also been detected in the Central Nervous System of cetaceans [10, 19–21, 24], although its significance still needs to be clarified [19–21, 24].

Although numerous cases of HV infection have been described in cetaceans in recent years, very few data is available concerning its detection in the digestive system of marine mammals [19]. Therefore, the description of HV-related lesions in the digestive tract extends our knowledge about HV in cetaceans and raises relevant questions about their transmission and significance. To the best of the authors’ knowledge, this is the first report of a proliferative lesion in the oropharyngeal and laryngopharyngeal mucosa associated with a GHV in a striped dolphin (*Stenella coeruleoalba*).

## Case presentation

**Fig. 1:**
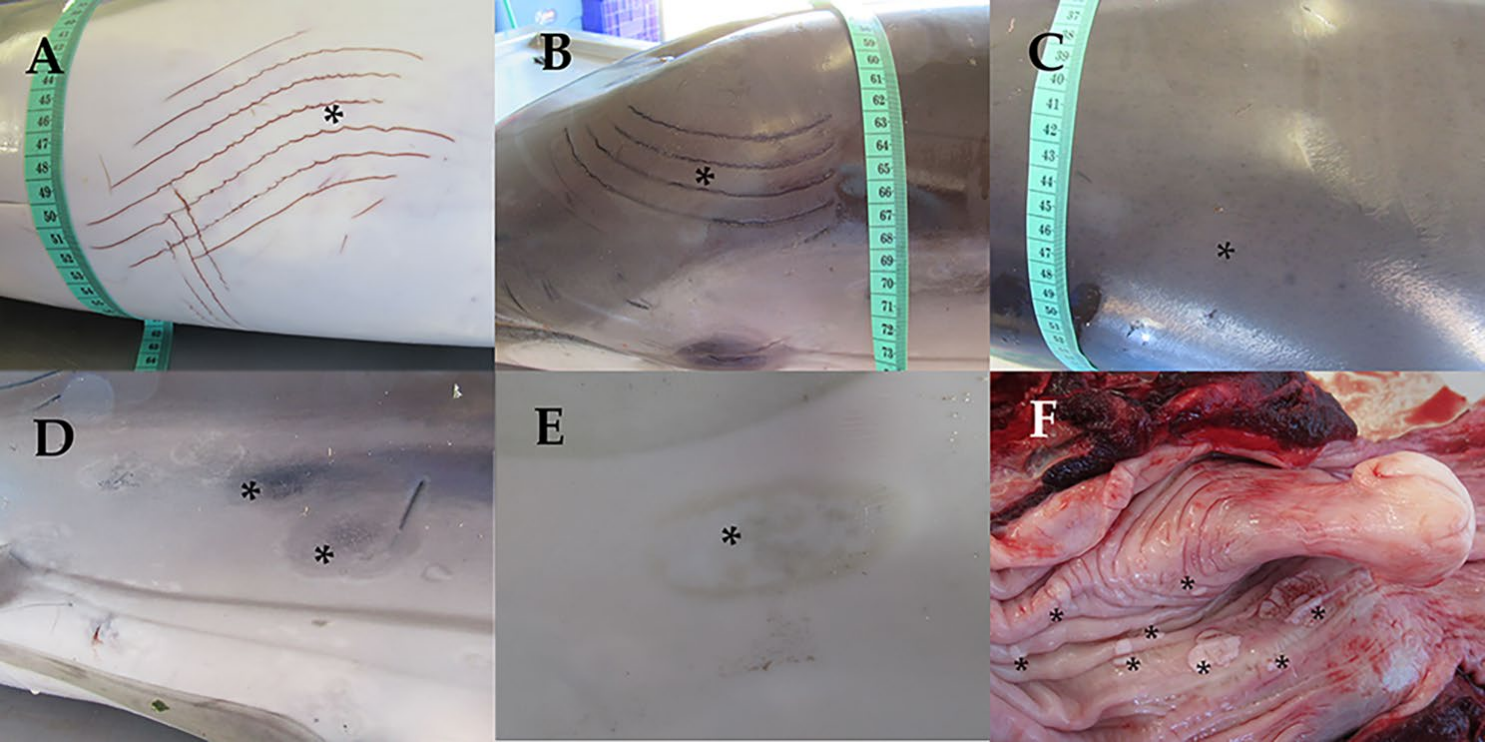
Most relevant macroscopic findings. The lesions described are highlighted by asterisks (*). **(A)** Rake marks in the abdominal region. **(B)** Rake marks in the head. **(C)** Diffuse hyper pigmented dotted lesion in the dorsal area of the thoracic region. **(D)** Oval lesions with irregular borders and hypo-hyper pigmented heterogeneous coloration in the left thoracic region. **(E)** Oval lesion with a hypo pigmented centre on the ventral part of the animal. **(F)** Oropharyngeal and laryngopharyngeal mucosa with multiple well-defined, whitish, irregularly shaped proliferative lesions (plaques)

On 6th August 2021, a juvenile male striped dolphin stranded alive at the shoreline of Pobla de Farnals, Valencian Community, Spain (39° 34’ 40’’ N, 0° 19’ 34’’ W), presenting difficulty maintaining balance and swimming in circles. The veterinary staff from Fundación Oceanogràfic, which is part of the Regional Stranding Network, carried out a complete veterinary examination including blood sampling. The animal was stabilized with emergency medication and the Rose Bengal Test (RBT) was performed for brucellosis screening in field conditions. Due to the animal condition, with neurological clinical signs, and a positive result in the RBT, the dolphin was humanely euthanized, and a full necropsy was performed the same day, according to previous literature [33, 34]. The main macroscopic lesions included longitudinal, parallel and shallow lacerations (rake marks) in the epidermis of the caudal fin, head, back and abdominal region (Fig. 1.A and 1.B); diffuse hyper pigmented dotting on the skin of the dorsal area of the thoracic region, cranial to the dorsal fin (Fig. 1.C); multifocal oval lesions with irregular borders, and heterogeneous hypo-hyperpigmented colour, in the left thoracic region (Fig. 1.D); oval lesion, approximately 4 cm in diameter, with a hypo pigmented centre on the ventral part of the animal (Fig. 1.E); presence of two specimens of *Xenobalanus* spp. on dorsal fin and right pectoral fin; emphysema in the central region of the left lung, with the presence of congestive areas medially and atelectasis in the most caudo-ventral area; hemorrhage with the presence of a clot in the middle dorsal zone of the left lung; moderate presence of free serosanguinous fluid in the pleural cavity; multiple, well-defined, whitish, raised, irregularly shaped, 0.3 to 4 cm, proliferative plaques on the oropharyngeal and laryngopharyngeal mucosa, adjacent to the glottis (Fig. 1.F); congestion, hemorrhage and edema in the pre scapular and pulmonary lymph nodes; and moderate congestion in the meninges.

Representative samples from several organs were collected during necropsy, including: normal skin, skin lesions, blubber, muscle, upper digestive mucosa with plaques, thyroid gland, adrenal gland, kidney, liver, pancreas, spleen, thymus, pharyngeal tonsils, pre scapular lymph node, mesenteric lymph node, lung, heart, spinal cord, cerebrum, cerebellum, cerebrospinal fluid (CSF) and serum.

Two sets of tissue samples were collected: the first set was preserved in 10% neutral buffered formalin and used for conventional histopathology, while the second set was stored at − 80 °C and used for molecular analysis.

**Fig. 2:**
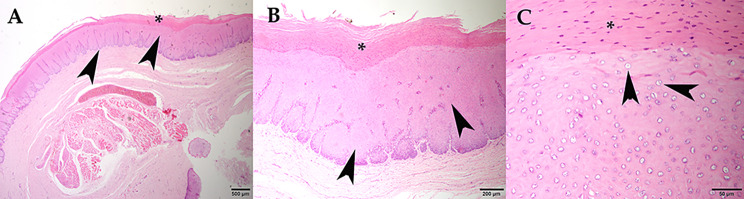
Raised plaque in the upper digestive mucosa. **A and B.** Epithelial hyperplasia (arrow) showing numerous layers piled over a folded and tortuous basement membrane and brightly eosinophilic, retained superficial layers (*). **C.** Detail of the superficial layers with hyperchromatic and occasionally fragmented nuclei (*) and underlying layers containing rare cells with marginated chromatin and a central, pale eosinophilic inclusion (arrow)

Histologically, the mucosa of the oropharyngeal and laryngopharyngeal plaques was thickened with over 80 layers piled over a folded and tortuous basement membrane. Basal cells were piled and disorganized with slightly basophilic cytoplasm and frequent mitotic figures. Middle layers contained mildly swollen cells with occasional perinuclear vacuolation. Superficial layers of approximately one third of the mucosal width were comprised of flattened cells with brightly eosinophilic cytoplasm and condensed, hyperchromatic, occasionally fragmented nuclei (Fig. 2A and B). Rare cells immediately underneath the hyper eosinophilic layers had a swollen nucleus with marginated hyperchromatic chromatin and a central pale eosinophilic inclusion (Fig. 2C).

Other histological findings, in order of significance, were: severe, chronic multifocal to coalescing, lymphoplasmacytic and histiocytic meningoencephalitis in the brainstem; confluent multifocal granulomatous pancreatic ductitis with intralesional trematodes; mild to moderate, chronic, confluent multifocal granulomatous pneumonia with intralesional 400 micron cross sections of nematodes with a celomic cavity, thick cuticle, celomyarian musculature, lateral cords, large intestine, consistent with metastrongyles (*Halocercus* sp.); subacute, multifocal, pulmonary lymph node sinus haemorrhage with erythrophagocytosis; mild, multifocal, suppurative and granulomatous cholangitis and multifocal bile stasis; granulomatous and eosinophilic mesenteric lymphadenitis; and moderate, multifocal, subcapsular renal haemorrhage.

For molecular diagnosis, tissue samples were evaluated for the presence of herpesvirus, CeMV and *Brucella* spp., as part of the health surveillance program carried out in the Valencian Community. In addition, the presence of poxvirus in skin lesions was ruled out. Standard precautions were taken during all laboratory procedures to avoid cross-contamination of samples. All samples were diluted 1:10 with phosphate-buffered saline (PBS) and homogenized using stainless steel 4.8-mm beads (Next Advance, New York, USA). Total DNA and RNA from the homogenates was extracted using the High Pure Viral Nucleic Acid Kit (Roche Diagnostics), based on the manufacturer’s instructions.

**Fig. 3:**
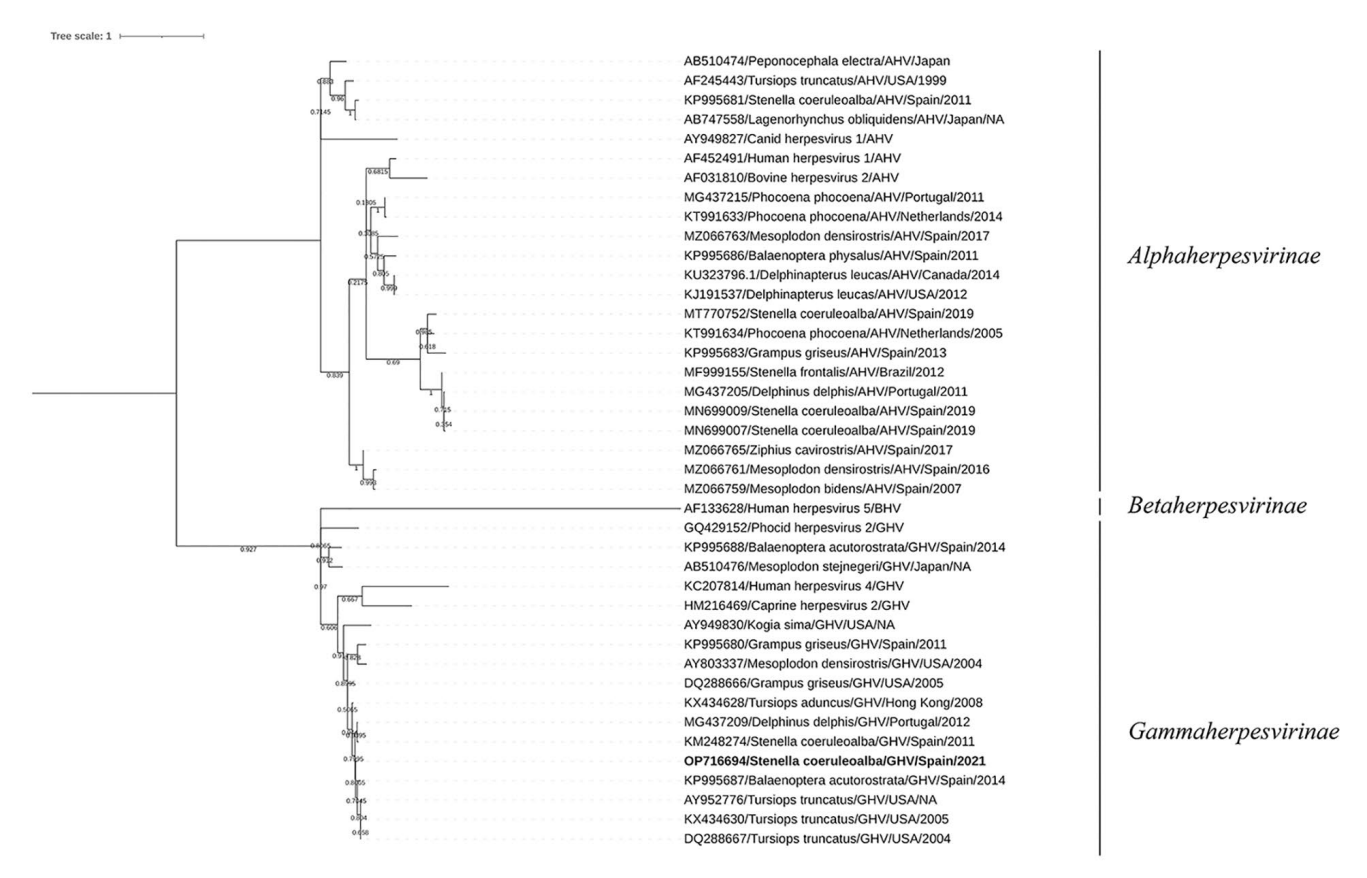
Maximum-likelihood phylogram of herpesviruses, based on partial nucleotide sequence of the DNApol gene. Nucleotide sequences of cetacean HV are named according to accession number, host species, subfamily of herpesvirus, country of origin and year of isolation. Sequence identified in the present study has been highlighted in bold and with a black square. Abbreviations: AHV Alphaherpesvirus, GHV Gammaherpesvirus.

The molecular diagnosis of the herpesvirus was carried out based on a previously described pan-herpesvirus nested PCR targeting the DNApol gene [35]. This assay was applied to the extracted DNA from skin, skin lesions, lesion in the upper digestive mucosa, pharyngeal tonsils, prescapular lymph node, cerebrum and cerebellum. Only the lesion lesion in the upper digestive mucosa was found positive. The 212-bp amplicon was purified using the QIAquick® Gel Extraction Kit (Qiagen, Hilden, Germany), and sequenced by Sanger sequencing. The obtained nucleotide sequence was submitted to Genbank and attributed the accession number OP716694.The herpesvirus nucleotide sequence was analyzed phylogenetically using the maximum likelihood method in MEGA X software [36] (Fig. 3). For this phylogenetic study, we included three human herpesvirus sequences, one canid herpesvirus sequence, one bovine herpesvirus sequence, one phocine herpesvirus sequence, one caprine herpesvirus sequence, and 33 cetacean herpesvirus sequences (including both odontocetes and mysticetes) detected in different in countries worldwide since 1999, in addition to the sequence described in this study. The accuracy of the sequence alignments was corrected in order to confer with the acceptance threshold value (< 0.8) for average p-distance [37, 38]. A bootstrap analysis with 2000 replicates was used to test the reliability of the tree. The resulting tree was rooted and edited with iTOL editor [39]. Nucleotide identities were calculated based on the p-distance (1 − p-distance)*100.

The phylogenetic analysis revealed that sequence OP716694 belong to the *Gammaherpesvirinae* subfamily and was part of a clade with a GHV from a striped dolphin from Spain (KM248274), a GHV from a short-beaked common dolphin (*Delphinus delphis*) from Portugal (MG437209), a GHV from a common minke whale (*Balaenoptera acutorostrata*) from Spain (KP995687), and GHV from bottlenose dolphins (*Tursiops truncatus*) from the USA (DQ288667, AY952776, KX434630).

Our sequence (OP716694) showed the highest nucleotide identity (98.13%) with GenBank sequence DQ288667, obtained from a penile lesion of a bottlenose dolphin from the USA. It also showed an identity higher than 90% with the following GenBank sequences: MG437209 (95.45%), obtained from a genital lesion of a short-beaked common dolphin from Portugal; KM248274 and KX434630 (93.94%), obtained from a lesion on the penis of a striped dolphin from Spain, and from a lesion on the pharyngeal mucosa of a bottlenose dolphin from the USA, respectively; KP995687 (93.55%), obtained from the skin of a common minke whale from Spain; KX434628 (93.46%), obtained from a penile lesion of an Indo-Pacific bottlenose dolphin (*Tursiops aduncus*) from Hong Kong; and AY952776 (92,42%), obtained from a penile lesion of a bottlenose dolphin from the USA.

RNA extracted from cerebrum, cerebellum, prescapular lymph node, lung, and pharyngeal tonsils were tested for CeMV using a reverse transcription-real time PCR assay targeting the fusion protein gene and is based on the Universal Probe Library platform [40]. All samples tested negative.

For *Brucella* spp. molecular detection was carried, following a previously described real-time PCR, targeting the IS711 insertion sequence [41], was performed on the following tissue samples: lung, pharyngeal tonsils, cerebrum, and CSF. The authors reported a lower limit of detection for this assay of 0.2-2 fg, depending on the Brucella species analyzed [41]. Since a negative result was considered when the Cycle threshold (Ct) was greater than 40, molecular positivity was observed in pharyngeal tonsils (Ct: 39.43) and CSF (Ct: 32.95). The PCR products from positive reactions were purified using the QIAquick® PCR Purification Kit (Qiagen, Hilden, Germany), following manufacturer’s instructions. The amplicons (178 bp [41]), were directly sequenced by Sanger DNA sequencing, which confirmed the positive result.

Additionally, the DNA from the skin lesions (n = 3) was tested for Poxvirus by conventional PCR, targeting DNApol gene [42]. However, molecular positivity was not detected.

## Discussion and conclusions

Detailed and reliable knowledge about the diseases and infectious agents that can affect cetaceans may provide useful insights into marine mammals’ health [19]. In this report, post-mortem analysis of a stranded striped dolphin and various routine molecular diagnosis were carried out to assess the lesions that the animal presented, as well as to implement a sanitary surveillance of the main infectious agents. This surveillance allowed us to associate a novel GHV sequence (OP716694) to a lesion in the oropharyngeal and laryngopharyngeal mucosa.

Other proliferative lesions in the oropharynx mucosa associated to GHV have been recently described in six rough-toothed dolphins (*Steno bredanensis*) from the Atlantic coast of the USA [43] and a Risso’s Dolphin (*Grampus griseus*) from the Mediterranean Spanish Coast [19]. GHV infections have been previously identified in hyperplastic skin and mucosal lesions, mainly genital [4, 9, 19, 24–27, 32, 44]. In line with this, the results of the phylogenetic analysis showed that the novel GHV sequence detected in this study was included in the GHV group and shared high identity with other GenBank sequences detected in skin (KP995687), pharyngeal (KX434630), and genital (DQ288667, MG437209, KM248274, KX434628, AY952776) lesions.

The infection of the upper digestive mucosa by a GHV sequence that shares significant identity with several genital sequences raises questions about its origin. Interestingly in a Risso’s dolphin from Spain, similar hyperplastic plaques were identified in both esophageal and genital mucosa, but they were associated with different HV subfamilies: GHV and Alphaherpesvirus (AHV), respectively [19]. Other similar GHV-associated esophageal lesions were detected in multiple rough-toothed dolphins of the same pod, suggesting that horizontal transmission may also exist [43]. Considering that in this case it was a juvenile dolphin, and that the present sequence shared 98.13% homology with the GenBank sequence DQ288667 detected in the genital mucosa, it is not possible to rule out suckling as a route of transmission since the proximity of the mammary slits to the genital slit in cetaceans. Other possible routes of infection involving orogenital contact include socio-sexual behaviors, such as ‘goosing’, in which an individual moves its rostrum into the genital area of another [45]. However, the role of water as a mechanical transmitter cannot be excluded either, as has been suggested for other HV [46, 47]. On the other hand, aerial transmission through desquamated infected cells from oropharyngeal lesions could be also a possibility. Respiratory and gastrointestinal systems are in close contact, [48] and this fact could imply that the GHV located in the esophageal mucosa could be excreted upon exhalation, and be potentially transmitted through the respiratory route.

Regarding the pathological significance of these oropharyngeal and laryngopharyngeal lesions, further research should be done in order to elucidate the transmission routes of the GHV that may be the cause of these lesions, as well as their impact on the health of the cetacean population.

Concerning the stranding cause, this dolphin had erratic swimming, probably due to the lymphoplasmacytic and histiocytic meningoencephalitis. This clinical sign, the location and type of lesion as well as the inflammatory infiltrate were consistent with neurobrucellosis [49]. Positive RBT and molecular analysis confirmed the *Brucella* spp. infection. Although bacterial culture is considered the ‘gold standard’ technique for the diagnosis of brucellosis, real-time PCR has also been previously performed to confirm Brucella infections in cetaceans [50, 51]. On the other hand, hemorrhages in the internal organs, the presence of the rake marks in the epidermis, and the separation of the parallel lacerations (interdental space) were suggestive of an aggressive interaction from bottlenose dolphins, as previously described in striped dolphins in this area [52, 53]. However, well-defined borders and the absence of fibrous tissue are indicative of an acute or subacute process. The other lesions were mainly associated with parasitic migrations.

It is not possible to conclude whether GHV infection occurred before or after *Brucella* infection. However, this individual exhibited signs of chronic illness, including poor body condition. Parasitic infestation, coupled with chronic brucellosis, are consistent with immunosuppression. It is well known that HV can establish latent infections [11, 12] and revert to an actively replicating state when an immunosuppressive or stress event occurs [13]. Therefore, it is likely that immunosuppression could have favored the appearance of this lesion or played a role. However, future studies which take these variables into account will need to be undertaken to better understand the pathogenesis of these lesions.

In conclusion, to our knowledge, we described for the first time a GHV-associated proliferative lesion in the upper digestive mucosa of a striped dolphin and we submitted the novel sequence to GenBank for future studies. The current findings enhance our understanding of herpesvirus pathology in cetaceans raising relevant questions about their transmission and significance.

## Data Availability

DNA sequence is available in the GenBank with following accession number: OP716694.

## Abbreviations

HV: Herpesvirus
DNApol: DNA polymerase
AHV: Alphaherpesvirus
GHV: Gammaherpesvirus
RBT: Rose Bengal Test
CSF: Cerebrospinal Fluid
CeMV: Cetacean Morbillivirus
Ct: Cycle threshold

## References

[1] Davison AJ, Eberle R, Ehlers B, Hayward GS, McGeoch DJ, Minson AC, Pellett PE, Roizman B, Studdert MJ, Thiry E (2009). The order Herpesvirales. Arch Virol.

[2] Davison AJ (2010). Herpesvirus systematics. Vet Microbiol.

[3] Noguchi K, Shimoda H, Terada Y, Shimojima M, Kohyama K, Inoshima Y, Maeda K (2013). Isolation of a novel herpesvirus from a Pacific white-sided dolphin. Arch Virol.

[4] Smolarek KA, Manire CA, Ewing RY, Saliki JT, Townsend FI, Ehlers B, Romero CH (2006). Identification of novel alpha- and gammaherpesviruses from cutaneous and mucosal lesions of dolphins and whales. J Virol Methods.

[5] Arbelo M, Sierra E, Esperón F, Watanabe TT, Bellière EN, Espinosa de los Monteros A, Fernández A (2010). Herpesvirus infection with severe lymphoid necrosis affecting a beaked whale stranded in the Canary Islands. Dis Aquat Organ.

[6] Sacristán C, Esperón F, Ewbank AC, Díaz-Delgado J, Ferreira-Machado E, Costa-Silva S, Sánchez-Sarmiento AM, Groch KR, Neves E, Pereira Dutra GH (2019). Novel herpesviruses in riverine and marine cetaceans from South America. Acta Trop.

[7] Miyoshi K, Nishida S, Sone E, Tajima Y, Makara M, Yoshioka M, Nakamura M, Yamada TK, Koike H (2011). Molecular Identification of Novel Alpha- and gammaherpesviruses from Cetaceans stranded on japanese coasts. Zoolog Sci.

[8] Bellehumeur C, Lair S, Romero CH, Provost C, Nielsen O, Gagnon CA (2015). Identification of a novel herpesvirus associated with a penile proliferative lesion in a beluga (*Delphinapterus leucas*). J Wildl Dis.

[9] van Beurden SJ, IJsseldijk LL, Ordonez SR, Förster C, de Vrieze G, Gröne A, Verheije MH, Kik M (2015). Identification of a novel gammaherpesvirus associated with (muco)cutaneous lesions in harbour porpoises (*Phocoena phocoena*). Arch Virol.

[10] Melero M, Crespo-Picazo JL, Rubio-Guerri C, García-Párraga D, Sánchez-Vizcaíno JM (2015). First molecular determination of herpesvirus from two mysticete species stranded in the Mediterranean Sea. BMC Vet Res.

[11] Speck SH, Ganem D (2010). Viral latency and its regulation: lessons from the gamma-herpesviruses. Cell Host Microbe.

[12] Penkert RR, Kalejta RF (2011). Tegument protein control of latent herpesvirus establishment and animation. Herpesviridae.

[13] Glaser RK-GJ. Stress-Associated Immune Modulation and Its Implications for Reactivation of Latent Herpesviruses. In: *Herpesvirus infections* edn. Edited by Glaser R JJ, editors. New York; 1994: 245–270.

[14] Flamand L, Gosselin J, Stefanescu I, Ablashi D, Menezes J (1995). Immunosuppressive effect of human herpesvirus 6 on T-cell functions: suppression of interleukin-2 synthesis and cell proliferation. Blood.

[15] Dagna L, Pritchett JC, Lusso P (2013). Immunomodulation and immunosuppression by human herpesvirus 6A and 6B. Future Virol.

[16] Babiuk LA, Lawman MJP, Griebel P. Immunosuppression by Bovine Herpesvirus 1 and Other Selected Herpesviruses. In: Virus-Induced Immunosuppression. edn. Edited by Specter S, Bendinelli M, Friedman H. Boston, MA: Springer US; 1989: 141–171.

[17] Hannant D, O’Neill T, Ostlund EN, Kydd JH, Hopkin PJ, Mumford JA (1999). Equid herpesvirus-induced immunosuppression is associated with lymphoid cells and not soluble circulating factors. Viral Immunol.

[18] Islam AF, Wong CW, Walkden-Brown SW, Colditz IG, Arzey KE, Groves PJ (2002). Immunosuppressive effects of Marek’s disease virus (MDV) and herpesvirus of turkeys (HVT) in broiler chickens and the protective effect of HVT vaccination against MDV challenge. Avian Pathol.

[19] Vargas-Castro I, Melero M, Crespo-Picazo JL, Jiménez MdlÁ, Sierra E, Rubio-Guerri C, Arbelo M, Fernández A, García-Párraga D (2021). Sánchez-Vizcaíno JM: systematic determination of Herpesvirus in Free-Ranging Cetaceans stranded in the western Mediterranean: tissue tropism and Associated Lesions. Viruses.

[20] Sierra E, Fernández A, Fernández-Maldonado C, Sacchini S, Felipe-Jiménez I, Segura-Göthlin S, Colom-Rivero A, Câmara N, Puig-Lozano R, Rambaldi AM et al. Molecular characterization of Herpesviral Encephalitis in Cetaceans: correlation with histopathological and immunohistochemical findings. Anim (Basel) 2022, 12(9). 10.3390/ani12091149 PMC9105563 35565575

[21] Giorda F, Crociara P, Iulini B, Gazzuola P, Favole A, Goria M, Serracca L, Dondo A, Crescio MI, Audino T (2022). Neuropathological characterization of Dolphin Morbillivirus infection in Cetaceans stranded in Italy. Animals.

[22] Sierra E, Fernández A, Felipe-Jiménez I, Zucca D, Díaz-Delgado J, Puig-Lozano R, Câmara N, Consoli F, Díaz-Santana P, Suárez-Santana C (2020). Histopathological Differential diagnosis of Meningoencephalitis in Cetaceans: Morbillivirus, Herpesvirus, Toxoplasma gondii, Brucella sp., and Nasitrema sp. Front veterinary Sci.

[23] Bellière EN, Esperón F, Arbelo M, Muñoz MJ, Fernández A, Sánchez-Vizcaíno JM (2010). Presence of herpesvirus in striped dolphins stranded during the cetacean morbillivirus epizootic along the Mediterranean Spanish coast in 2007. Arch Virol.

[24] Vargas-Castro I, Crespo-Picazo JL, Rivera-Arroyo B, Sánchez R, Marco-Cabedo V, Jiménez-Martínez M, Fayos M, Serdio Á, García-Párraga D, Sánchez-Vizcaíno JM (2020). Alpha- and gammaherpesviruses in stranded striped dolphins (*Stenella coeruleoalba*) from Spain: first molecular detection of gammaherpesvirus infection in central nervous system of odontocetes. BMC Vet Res.

[25] Saliki JT, Cooper EJ, Rotstein DS, Caseltine SL, Pabst DA, McLellan WA, Govett P, Harms C, Smolarek KA, Romero CH (2006). A novel gammaherpesvirus associated with genital lesions in a Blainville’s beaked whale (*Mesoplodon densirostris*). J Wildl Dis.

[26] van Elk CE, van de Bildt MW, de Jong AA, Osterhaus AD, Kuiken T (2009). Genital herpesvirus in bottlenose dolphins (*Tursiops truncatus*): cultivation, epidemiology and associated pathology. J Wildl Dis.

[27] Sierra E, Díaz-Delgado J, Arbelo M, Andrada M, Sacchini S, Fernández A (2015). Herpesvirus-associated genital lesions in a stranded striped dolphin (*Stenella coeruleoalba*) in the Canary Islands, Spain. J Wildl Dis.

[28] Lecis R, Tocchetti M, Rotta A, Naitana S, Ganges L, Pittau M, Alberti A (2014). First gammaherpesvirus detection in a free-living Mediterranean bottlenose dolphin. J Zoo Wildl Med.

[29] Bento MC, Canha R, Eira C, Vingada J, Nicolau L, Ferreira M, Domingo M, Tavares L, Duarte A (2019). Herpesvirus infection in marine mammals: a retrospective molecular survey of stranded cetaceans in the portuguese coastline. Infect Genet Evol.

[30] Rehtanz M, Bossart GD, Fair PA, Reif JS, Ghim S-j, Jenson AB (2012). Papillomaviruses and herpesviruses: who is who in genital tumor development of free-ranging Atlantic bottlenose dolphins (*Tursiops truncatus*)?. Vet Microbiol.

[31] Seade GCC, Cerqueira VD, Sierra E, Chaves JF, Moura MAO, Montão DP, Riet-Correa G, Oliveira CA, Siciliano S, Emin-Lima R (2017). Herpesviral infection in a Guiana dolphin (*Sotalia guianensis*) from the northern coast of Brazil. J Vet Diagn Invest.

[32] van Elk C, van de Bildt M, van Run P, de Jong A, Getu S, Verjans G, Osterhaus A, Kuiken T (2016). Central nervous system disease and genital disease in harbor porpoises (*Phocoena phocoena*) are associated with different herpesviruses. Vet Res.

[33] Geraci J, Lounsbury V. Marine mammals ashore: a field guide for strandings. In., edn.: National Aquarium in Baltimore; 2005.

[34] Kuiken T, Hartmann MG. Cetacean dissection techniques and tissue sampling. ECS Newsletter: Special Issue 1991:1–39.

[35] van Devanter DR, Warrener P, Bennett L, Schultz ER, Coulter S, Garber RL, Rose TM (1996). Detection and analysis of diverse herpesviral species by consensus primer PCR. J Clin Microbiol.

[36] Kumar S, Stecher G, Li M, Knyaz C, Tamura K (2018). MEGA X: Molecular Evolutionary Genetics Analysis across Computing Platforms. Mol Biol Evol.

[37] Thompson JD, Plewniak F, Poch O (1999). A comprehensive comparison of multiple sequence alignment programs. Nucleic Acids Res.

[38] Ogden TH, Rosenberg MS (2006). Multiple sequence alignment accuracy and phylogenetic inference. Syst Biol.

[39] Letunic I, Bork P (2021). Interactive tree of life (iTOL) v5: an online tool for phylogenetic tree display and annotation. Nucleic Acids Res.

[40] Rubio-Guerri C, Melero M, Rivera-Arroyo B, Bellière EN, Crespo JL, García-Párraga D, Esperón F, Sánchez-Vizcaíno JM (2013). Simultaneous diagnosis of Cetacean morbillivirus infection in dolphins stranded in the spanish Mediterranean sea in 2011 using a novel Universal Probe Library (UPL) RT-PCR assay. Vet Microbiol.

[41] Bounaadja L, Albert D, Chénais B, Hénault S, Zygmunt MS, Poliak S, Garin-Bastuji B (2009). Real-time PCR for identification of *Brucella* spp.: a comparative study of IS711, bcsp31 and per target genes. Vet Microbiol.

[42] Bracht AJ, Brudek RL, Ewing RY, Manire CA, Burek KA, Rosa C, Beckmen KB, Maruniak JE, Romero CH (2006). Genetic identification of novel poxviruses of cetaceans and pinnipeds. Arch Virol.

[43] Ewing RY, Rotstein DS, McLellan WA, Costidis AM, Lovewell G, Schaefer AM, Romero CH, Bossart GD. Macroscopic and histopathologic findings from a Mass Stranding of Rough-Toothed Dolphins (*Steno bredanensis*) in 2005 on Marathon Key, Florida, USA. Front Veterinary Sci 2020, 7(572). 10.3389/fvets.2020.00572 PMC7492606 32984413

[44] Elk CEv B, MWGvd J, AAWd, Osterhaus ADME, Kuiken T (2009). Genital herpesvirus in bottlenose dolphins (*Tursiops truncatus*): cultivation, epidemiology, and associated pathology. J Wildl Dis.

[45] Mann J, Connor RC, Tyack PL, Whitehead H. Cetacean societies: field studies of dolphins and whales. University of Chicago Press; 2000.

[46] Ewbank AC, Duarte-Benvenuto A, Zamana-Ramblas R, Sacristán I, Costa-Silva S, Carvalho VL, de Mello DMD, da Silva VMF, Catão-Dias JL, Sacristán C (2023). Herpesvirus and adenovirus surveillance in threatened wild West Indian (*Trichechus manatus*) and amazonian manatees (*Trichechus inunguis*), Brazil. Acta Trop.

[47] Dayaram A, Franz M, Schattschneider A, Damiani AM, Bischofberger S, Osterrieder N, Greenwood AD (2017). Long term stability and infectivity of herpesviruses in water. Sci Rep.

[48] Varela RA, Schmidt K, Goldstein JD, Bossart GD (2007). Evaluation of Cetacean and Sirenian Cytologic samples. Veterinary Clin North America: Exotic Anim Pract.

[49] Guzmán-Verri C, González-Barrientos R, Hernández-Mora G, Morales J-A, Baquero-Calvo E, Chaves-Olarte E, Moreno E (2012). *Brucella ceti* and brucellosis in cetaceans. Front Cell Infect Microbiol.

[50] Sierra E, Fernández A, Felipe-Jiménez I, Zucca D, Di Francesco G, Díaz-Delgado J, Sacchini S, Rivero MA, Arbelo M (2019). Neurobrucellosis in a common bottlenose dolphin (*Tursiops truncatus*) stranded in the Canary Islands. BMC Vet Res.

[51] Venn-Watson S, Colegrove KM, Litz J, Kinsel M, Terio K, Saliki J, Fire S, Carmichael R, Chevis C, Hatchett W (2015). Adrenal gland and lung lesions in Gulf of Mexico common bottlenose dolphins (*Tursiops truncatus*) found dead following the Deepwater Horizon oil spill. PLoS ONE.

[52] Crespo-Picazo JL, Rubio-Guerri C, Jiménez MA, Aznar FJ, Marco-Cabedo V, Melero M, Sánchez-Vizcaíno JM, Gozalbes P (2021). García-Párraga D: Bottlenose dolphins (*Tursiops truncatus*) aggressive behavior towards other cetacean species in the western Mediterranean. Sci Rep.

[53] Barnett J, Davison N, Deaville R, Monies R, Loveridge J, Tregenza N, Jepson PD (2009). Postmortem evidence of interactions of bottlenose dolphins (*Tursiops truncatus*) with other dolphin species in south-west England. Vet Rec.

